# Clinical Significance of Low-Density Granulocytes in Acute Pancreatitis

**DOI:** 10.1155/mi/5275081

**Published:** 2025-07-10

**Authors:** Jiren Wang, Xinwen Chen, Youli Ke, Nannan Zhu, Jiejie Zhu, Hao Ding, Linlin Pan, Chenao Zhang, Xingyu Liu, Yumei Wu, Huizhong Gan, Qiao Mei

**Affiliations:** ^1^Department of Gastroenterology, The First Affiliated Hospital of Anhui Medical University, Hefei City, Anhui Province, China; ^2^Department of Gastroenterology, Hefei City First People's Hospital, Hefei City, Anhui Province, China

**Keywords:** acute pancreatitis, LDGs, PBMCs

## Abstract

**Background:** Low-density granulocytes (LDGs), a unique subset of neutrophils, have been shown in previous studies to exhibit distinct biological functions in immune-inflammatory diseases. However, their role in acute pancreatitis (AP) remains unclear.

**Objective:** To investigate the association between peripheral blood LDGs levels and clinical severity in patients with AP.

**Methods:** This cross-sectional study analyzed peripheral LDGs levels in 41 mild AP (MAP) patients, 40 moderate-to-severe AP (SAP) patients, and 41 healthy controls (HC). LDGs were measured within 24 h of admission. Statistical analysis, including Kruskal–Wallis tests and logistic regression, assessed LDGs' association with clinical characteristics and AP severity.

**Results:** LDGs levels were significantly higher in moderate-to-SAP compared to MAP and HC (*p* < 0.001). Elevated LDGs levels correlated positively with white blood cell count (WBC), glucose (GLU), neutrophil-to-lymphocyte ratio, Acute Physiology and Chronic Health Evaluation II (APACHE II), and Bedside Index for Severity in Acute Pancreatitis (BISAP) scores (*p* < 0.05). Logistic regression identified LDGs as an independent risk factor for moderate-to-SAP. Receiver operating characteristic (ROC) analysis revealed high predictive accuracy, the area under the curve (AUC = 0.967) at a 2.96% cutoff, with 95.0% sensitivity and 90.2% specificity. The follow-up indicated that patients with LDGs > 2.96% had a higher risk of recurrence.

**Conclusion:** Elevated peripheral LDGs levels in AP patients are associated with disease activity.

## 1. Introduction

Acute pancreatitis (AP) is a common clinical condition characterized primarily by abdominal pain, with its pathogenesis linked to pancreatic autodigestion and inflammatory responses. In recent years, the global incidence of AP has been steadily increasing. The imaging diagnosis of AP mainly relies on magnetic resonance imaging (MRI), endoscopic ultrasound (EUS) [1, 2]. The revised Atlanta classification categorizes AP severity into three levels: mild, moderate, and severe [3]. Mild AP (MAP) is typically self-limiting with a favorable prognosis. However, ~20% of patients experience an early systemic inflammatory response syndrome (SIRS) triggered by pro-inflammatory immune cells, which can progress to severe AP (SAP) and significantly increase mortality [4, 5]. Therefore, accurate assessment of AP severity is critical. Inflammatory cells play a central role in the pathogenesis of AP, contributing to pancreatic tissue damage, local necrosis, and infections in the pancreatic microenvironment. Systemically, inflammatory cells release large quantities of inflammatory mediators into circulation, potentially leading to multiple organ dysfunction syndrome (MODS). Understanding the mechanisms by which different inflammatory cells act is essential for developing effective strategies to prevent and manage the inflammatory cascade associated with AP.

Neutrophils, a key component of the innate immune system, have been extensively studied and shown to play a destructive role in the progression of AP. By releasing various inflammatory mediators, neutrophils exacerbate local inflammation [6], causing pancreatic cell injury and necrosis, which further aggravates SIRS and distant organ damage [7, 8]. Experimental models of bile acid-induced AP in mice have demonstrated that neutrophils also contribute to pancreatic destruction through the release of neutrophil extracellular traps (NETs) [9]. Low-density granulocytes (LDGs), a unique subset of neutrophils, have been identified as originating from the neutrophil lineage [10]. Studies have shown that LDGs play a pro-inflammatory role in autoimmune diseases by inducing T cells to produce inflammatory cytokines, such as interferon-gamma (IFN-γ) and tumor necrosis factor-alpha (TNF-α) [11]. Additionally, LDGs are closely associated with NET formation [12]. In contrast, in sepsis, LDGs exhibit immunosuppressive effects, increasing the risk of secondary hospital-acquired infections in septic patients [13, 14].These findings suggest that LDGs exhibit distinct biological functions in different inflammatory diseases.

Currently, no studies have investigated the relationship between LDGs levels and AP. Therefore, this study aims to explore the association between LDGs levels and the clinical course of AP by measuring LDGs levels in AP patients and analyzing their potential role in disease progression.

## 2. Materials and Methods

### 2.1. Patients Enrolled

Peripheral blood samples were collected from AP patients hospitalized at our institution and from healthy controls (HC) in the surrounding population. AP diagnosis was based on the Atlanta diagnostic criteria, requiring at least two of the following three features: (1) abdominal pain consistent with AP; (2) serum amylase or lipase levels at least three times the upper limit of normal; (3) imaging evidence of pancreatic injury. The severity of AP was classified into three categories: (1) MAP: No organ failure and no local complications; (2) Moderately SAP: Transient organ failure (≤ 48 h) or the presence of local complications; (3) SAP: Persistent organ failure (> 48 h) [3].

Exclusion criteria included: (1) age < 18 years or > 80 years; (2) pancreatic tumors; (3) acute exacerbation of chronic pancreatitis; (4) autoimmune diseases; (5) severe preexisting cardiac or pulmonary conditions; (6) active infections; the normal control group consisted of individuals aged 18–80 years who were undergoing routine health examinations, had no history of underlying diseases, and met the same exclusion criteria as AP patients. Ultimately, 81 AP patients were included in the analysis, comprising 41 MAP patients and 40 patients with moderately-severe to SAP. All procedures were conducted in accordance with relevant regulations and guidelines and adhered to the principles of the Declaration of Helsinki (Approval No.: PJ 2024-05-65).

The demographic and clinical information of AP patients, including gender, age, length of hospital stay, hypertension, diabetes, hyperlipidemia, gallstones, and relevant laboratory parameters, were systematically collected and organized. These clinical indicators were derived from laboratory tests conducted within 24 h of admission. Additionally, AP patients were scored using the Bedside Index for Severity in Acute Pancreatitis (BISAP) score, Acute Physiology and Chronic Health Evaluation II (APACHE II) score.

The APACHE II and BISAP scores are used to evaluate the severity of illness in patients with AP. The APACHE II score is calculated based on acute physiological parameters, age, and chronic health status, with a total score ranging from 0 to 71. The BISAP score is determined using five clinical indicators: blood urea nitrogen (BUN), mental status, SIRS, age over 60 years, and the presence of pleural effusion, with a score range of 0–5. Higher scores on either scale indicate increased disease severity and a higher risk of mortality [15, 16]. For detailed information, please refer to (Supporting Information 1).

### 2.2. Quantification of Circulating LDGs

Flow cytometry was used to measure the levels of LDGs in peripheral blood within 24 h of admission for AP patients and in HC. Peripheral blood was collected into EDTA tubes, and peripheral blood mononuclear cells (PBMCs) were isolated using Ficoll–Paque (TBD, Tianjin, China) density gradient centrifugation. The PBMCs were initially treated with red blood cell lysis buffer, followed by washing with PBS and MASC-Buffer.

To prepare the cell suspension, 3 µL of CD14 antibody and CD15 antibody were added (APC antihuman CD14, clone M5E2, Biolegend; TIFC antihuman CD15, clone W6D3, Biolegend), and the mixture was incubated in the dark for 20 min. The cells were then resuspended in MASC-Buffer and stained with 7 µL of 4′,6-diamidino-2-phenylindole (DAPI) (catalog 422801, Biolegend) for fluorescence analysis. LDGs were identified within the PBMCs population as CD14^low^/CD15^+^ cells and quantified by flow cytometry, CytoFlex (Beckman Coulter, San Diego, CA, United States) [17]. The proportion of LDGs in the peripheral blood of AP patients and HC was determined using this method.

### 2.3. Statistical Methodology

(Supporting Information 2) Statistical analyses were conducted using SPSS (version 26.0), GraphPad Prism (version 10.1.2), and R (version 4.2.2), with the results visualized through these tools. Continuous variables following a normal distribution were expressed as mean ± standard deviation (SD) and compared between groups using the Student's *t*-test. Non-normally distributed variables were presented as median (Q1, Q3), with comparisons between two groups performed using the Mann–Whitney *U* test, and comparisons across multiple groups conducted using the Kruskal–Wallis test. Post hoc pairwise comparisons for nonparametric data were also performed.

Categorical variables were expressed as percentages and compared using the chi-square test (*χ*^*2*^ test). Correlation analyses were conducted using Spearman's rank correlation test. *p*-Value < 0.05 was considered statistically significant.

## 3. Result

### 3.1. Baseline Data of AP Patients

A total of 81 patients with AP were included in this study, comprising 41 cases of MAP and 40 cases of moderate to SAP, along with 41 HC. LDGs levels were measured, and relevant clinical data were collected. The median age of AP patients was 46.0 years (35.0, 58.0), with males accounting for 56.1% of the cohort.

Hospital length of stay differed significantly between MAP and moderate-to-SAP patients (MAP: 7 [6, 9] days; moderate-to-SAP: 11 [9, 17] days, *p* < 0.001). Significant differences were also observed between the two AP groups in hospital stay duration, NEUT, C-reactive protein (CRP), glucose (GLU), albumin (ALB), potassium (K), and calcium (Ca) levels (*p* < 0.05). No significant differences were found for other clinical parameters (Table 1).

### 3.2. Comparison of LDGs Levels Between AP Patients and HC

**Figure 1:**
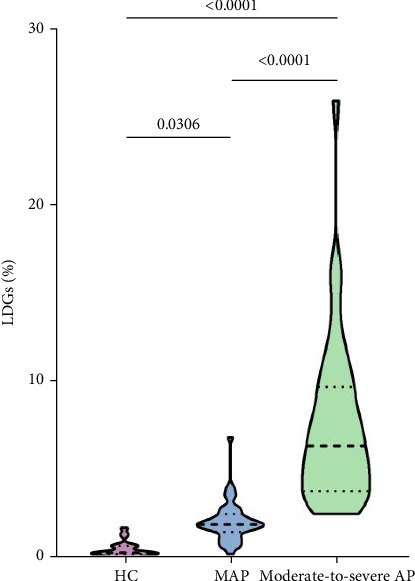
Among-group comparison of LDGs levels in different degrees of acute pancreatitis and healthy controls.

1. Peripheral blood LDGs levels were measured in HC and AP patients using flow cytometry. Representative results are shown in (Supporting Information 3), where the gating strategy was performed using FlowJo. LDGs were identified as the CD14^low/⁻^CD15^+^ cell population. The peripheral blood LDGs levels in AP patients were significantly higher than those in HC (Supporting Information 3).
2. A comparison of LDGs levels among AP patients with different severity was performed using the Kruskal–Wallis test, which revealed a significant difference between the MAP and moderate-to-SAP groups (*p* < 0.05). Moreover, LDGs levels differed significantly across the HC, MAP, and moderate-to-SAP groups (MAP: 1.96 [1.60, 2.52]; moderate-to-SAP: 5.69 [3.58, 8.88]; [*p* < 0.001]) (Figure 1.)

### 3.3. Correlation Analysis Between LDGs Levels and Clinical Characteristics in AP Patients

The clinical characteristics, etiologies, and comorbidities of 81 AP patients were analyzed, including hyperlipidemia, gallstones, alcohol consumption, diabetes, and local complications. Local complications encompassed peripancreatic fluid collections, necrotic collections, walled-off necrosis, and pancreatic pseudocyst formation [3]. To evaluate the potential influence of these factors on LDGs levels, the Mann–Whitney *U* test was performed. The results indicated that LDGs levels were significantly associated with local complications but showed no association with alcohol consumption, gallstones, diabetes, hyperlipidemia, or intensive care unit (ICU) admission.

Among patients with moderate-to-SAP, a comparative analysis revealed no significant differences in LDGs levels based on the presence or absence of SIRS (SIRS, *n* = 37; without SIRS, *n* = 3) or organ failure (with organ failure, *n* = 27; without organ failure, *n* = 13) (SIRS, *p*=0.296; organ failure, *p*=0.217)

Spearman correlation analysis revealed that LDGs levels in AP patients were positively correlated with white blood cell count (WBC), GLU, neutrophil-to-lymphocyte ratio (NLR), APACHE II score, and BISAP score (*p* < 0.05 for all). Conversely, LDGs levels were negatively correlated with serum calcium (*p* < 0.05). No significant correlations were observed between LDGs levels and platelet count (PLT), CRP, ALB, TBIL, alanine aminotransferase (ALT), BUN, K, or body mass index (BMI) (*p* > 0.05) (Supporting Information 4).

### 3.4. Univariate and Multivariate Logistic Regression Analysis of Clinical Characteristics in Moderate to SAP

Univariate logistic regression analysis indicated that LDGs levels, hospital stay duration, NEUT, and CRP were positively associated with the severity of inflammation in AP.

Multivariate logistic regression analysis further demonstrated that elevated LDGs levels were a significant independent predictor of progression to moderate-to-SAP (OR = 6.36; 95% CI: 2.16–18.68; *p*=0.001). CRP also played a significant role in predicting AP severity (OR = 1.02; 95% CI: 1.00–1.05; *p*=0.034) (Table 2).

### 3.5. Efficacy of LDGs in Assessing the Severity of AP

**Figure 2:**
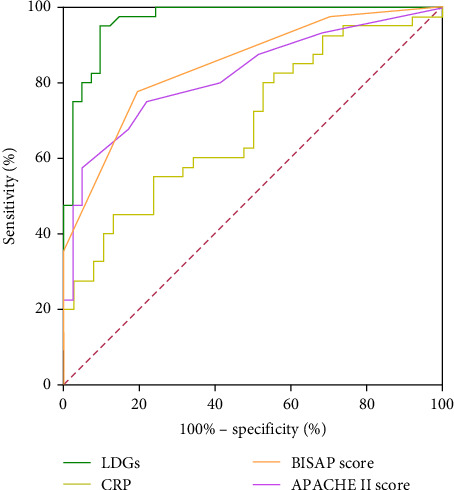
Receiver operating characteristics curves for four related indicators.

In AP patients, the area under the curve (AUC) values for LDGs levels, CRP, APACHE II score, and BISAP score were 0.967, 0.686, 0.821, and 0.845, respectively. At a cutoff value of 2.96%, LDGs levels demonstrated a sensitivity of 95.0% and a specificity of 90.2% for predicting AP severity (Table 3, Figure 2).

### 3.6. Follow-Up Analysis

A follow-up investigation was conducted for 81 AP patients after discharge. Patients who experienced AP recurrence or death were classified as having a poor prognosis, while those with improved findings on follow-up contrast-enhanced abdominal CT and no recurrence were classified as having a favorable prognosis. Four patients were lost to follow-up (loss rate = 4.9%) and excluded from the analysis.

**Figure 3:**
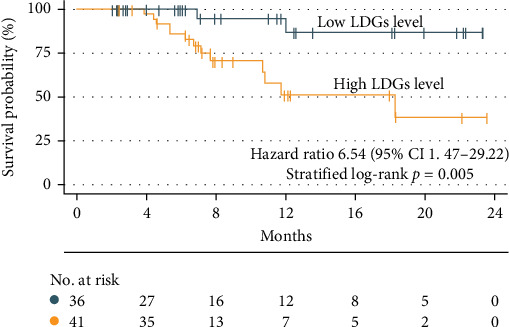
Survival curves for two different LDGs levels.

Using the optimal LDGs cutoff value of 2.96%, univariate Cox regression analysis revealed that LDGs grade was significantly associated with prognosis (*p*=0.014). Patients with high LDGs levels were at a greater risk of recurrence or death (OR = 6.54; 95% CI: 1.47–29.22). Kaplan–Meier (K–M) survival curves were subsequently constructed, demonstrating that patients with high LDGs levels had an increased risk of recurrence and a progressive decline in survival as time from discharge increased (log-rank test, *p*=0.005) (Figure 3).

## 4. Discussion

AP is an inflammatory disease characterized by pancreatic injury and autodigestion, with neutrophil infiltration into pancreatic parenchymal cells playing a key role in its pathogenesis [18]. Once activated at the site of inflammation, neutrophils cause persistent tissue damage [11]. Damaged acinar cells release damage-associated molecular patterns (DAMPs), further recruiting and activating neutrophils, amplifying inflammation, and damaging other organs [19, 20]. This cascade contributes to the progression of pancreatitis and the development of SIRS.

Studies have shown that LDGs, a specialized subset of neutrophils, are closely associated with the activity of various chronic inflammatory diseases [10, 14, 21]. In systemic lupus erythematosus (SLE), LDGs exhibit a strong pro-inflammatory role by inducing T cells to release IFN-γ, TNF-α, and lymphotoxin alpha (LTA) [22]. Conversely, in infectious diseases, LDGs can suppress T-cell proliferation and differentiation, and a subset of LDGs may modulate inflammation and balance immune responses [23]. Furthermore, LDGs can spontaneously form neutrophil extracellular traps NETs via mitochondrial ROS [24–26], suggesting that LDGs represent a unique pro-inflammatory neutrophil subset that may contribute to pancreatic damage [27]. However, no prior studies have established a correlation between LDGs and AP.

This study is the first to investigate the relationship between LDGs levels and AP. Using flow cytometry, we measured peripheral blood LDGs levels in AP patients and HC. For the first time, we demonstrated that LDGs levels are significantly elevated in the peripheral blood of AP patients compared to HC. Due to the limited number of moderate and SAP cases, these groups were combined into a “moderate to SAP” category for analysis. Notably, LDGs levels differed significantly between the MAP and moderate to SAP groups and between each AP group and the HC group, findings that we found particularly exciting.

To evaluate the predictive value of LDGs in assessing AP severity, we performed receiver operating characteristic (ROC) curve analysis for LDGs, CRP, APACHE II score, and BISAP score. The AUC values for CRP, APACHE II score, and BISAP score were 0.686, 0.821, and 0.845, respectively, consistent with previous findings that these indicators have strong predictive value for AP severity [28–30]. However, LDGs demonstrated superior sensitivity and specificity for predicting AP activity, with an AUC of 0.967.

Univariate and multivariate logistic regression analyses were conducted to explore the impact of LDGs levels on AP severity. The results confirmed that LDGs levels were significantly associated with AP severity and served as an independent risk factor. Furthermore, our study found that LDGs levels were not associated with comorbidities, such as hyperlipidemia, gallstones, alcohol consumption, diabetes, or ICU admission, but were significantly correlated with local complications. According to the Atlanta classification, local complications are a key diagnostic criterion for moderate AP severity [3]. Our findings revealed that higher LDGs levels were associated with increased inflammation and a greater likelihood of local complications, explaining the correlation between LDGs and AP severity.

Additionally, LDGs levels were positively correlated with certain laboratory markers and inflammatory indices, including WBC, GLU, NLR, APACHE II score, and BISAP score, and negatively correlated with serum calcium.

LDGs did not demonstrate significant impact in assessing the presence of SIRS and organ failure in patients with moderate to severe pancreatitis. This may be related to the insufficient total number of cases included (moderate to SAP, *n* = 40). We plan to further collect more cases to analyze the correlation.

A follow-up analysis of 77 patients revealed that while LDGs levels did not directly affect AP recurrence, patient survival rates differed significantly when stratified by LDGs levels using the optimal cutoff value.

In this study, LDGs demonstrated an AUC of 0.967 for assessing the severity of AP, markedly outperforming conventional indicators, such as CRP (AUC = 0.686), APACHE II (AUC = 0.821), and BISAP (AUC = 0.845). LDGs exhibited a sensitivity of 95.0% and a specificity of 90.2%. The high sensitivity supports early identification of SAP, reducing the risk of delayed intervention, while the high specificity helps avoid unnecessary procedures and resource overuse. These findings suggest that LDGs significantly reduce both false-negative and false-positive outcomes, underscoring their excellent diagnostic precision.

Currently, widely used clinical tools for predicting AP severity include CRP, NLR, APACHE II, and BISAP scores [31, 32]. However, each has notable limitations: CRP levels typically elevate only 24–48 h after symptom onset; NLR is highly susceptible to infection-related fluctuations; APACHE II is complex and subject to clinician interpretation; and although BISAP is user-friendly, its early predictive capacity remains limited. Consequently, there is a critical need for a rapid, sensitive, and highly specific early biomarker.

LDGs, a distinct neutrophil subset, have demonstrated robust diagnostic and prognostic value in various inflammatory conditions, including SLE, rheumatoid arthritis (RA), and sepsis. Our findings reveal that LDGs levels are significantly elevated in AP and strongly correlated with disease severity. This highlights their potential as a novel, independent, and reliable early biomarker for clinical application, offering a promising new strategy for the accurate assessment of AP severity.

In conclusion, our study demonstrates that LDGs levels in the peripheral blood of AP patients are markedly elevated compared to HC. LDGs levels can serve as a reliable marker to predict AP activity and assess the risk of recurrence.

## Figures and Tables

**Table 1:** Demographic, clinical, and laboratory characteristics of study participants (*n* = 122).

Variables	HC (*n* = 41)	MAP (*n* = 41)	Moderate-to-severe AP (*n* = 40)	*p*-Value
Male (*n*%)	20 (48.8)	22 (53.7)	23 (57.5)	0.733
Age (years)	42.0 (28.5, 49.5)	49.0 (36.0, 59.5)	45.5 (35, 64)	0.079
Hospital day (day)	—	7 (6, 9)	11 (9, 17)	<0.001
BMI (kg/m^2^)	—	24.4 ± 3.5	24.8 ± 3.1	0.176
LDGs (%)	0.30 (0.21, 0.68)	1.96 (1.60, 2.52)	5.69 (3.58, 8.88)	<0.001
NEUT (× 10^9^/L)	—	8.73 ± 4.08	10.9 ± 4.9	0.027
CRP (mg/L)	—	52.46 (4.46, 128.16)	111.82 (26.29, 167.56)	0.004
Glu (mmol/L)	—	5.71 (4.77, 7.66)	7.85 (6.30, 12.79)	0.006
ALB (g/L)	—	43.48 ± 5.32	39.50 ± 6.67	0.008
ALT (U/L)	—	44.50 (22.85, 85.23)	29.10 (19.00, 60.00)	0.253
AST (U/L)	—	30.20 (18.23, 70.18)	32.70 (25.00, 71.40)	0.637
ALP (U/L)	—	94.25 (78.05, 133.75)	95.00 (73.90, 159.90)	0.574
GGT (U/L)	—	48.00 (27.98, 181.33)	70.00 (34.00, 170.40)	0.770
TBIL (μmol/L)	—	18.49 (12.17, 27.86)	19.16 (12.43, 27.44)	0.828
DBIL (μmol/L)	—	3.67 (2.64, 7.82)	5.78 (2.73, 10.76)	0.396
BUN (μmol/L)	—	4.29 (3.41, 5.30)	5.27 (3.89, 6.86)	0.090
CRE (μmol/L)	—	59.40 (46.78, 64.58)	58.40 (47.40, 70.90)	0.861
K (mmol/L)	—	4.06 (3.81, 4.22)	3.80 (3.60, 4.14)	0.043
Ca (mmol/L)	—	2.24 (2.14, 2.35)	2.07 (1.98, 2.22)	0.001
High blood, yes = *n* (%)	—	8 (19.5)	14 (35.0)	—
Diabetes, yes = *n* (%)	—	11 (26.8)	12 (30.0)	—
Cholelithiasis, yes = *n* (%)	—	13 (31.7)	17 (42.5)	—
Hyperlipidemia, yes = *n* (%)	—	15 (36.6)	17 (42.5)	—
SIRS, yes = *n* (%)	—	—	37 (92.5)	—
Organ failure, yes = *n* (%)	—	—	27 (67.5)	—

Abbreviations: ALB, albumin; ALP, alkaline phosphatase; ALT, alanine aminotransferase; AST, aspartate aminotransferase; BMI, body mass index; BUN, blood urea nitrogen; Ca, calcium; CRE, creatinine; CRP, C-reactive protein; DBIL, direct bilirubin; GGT, γ-glutamyl transpeptidase; GLU, glucose; K, potassium; LDGs, low-density granulocytes; NEUT, neutrophil count; SIRS, systemic inflammatory response syndrome; TBIL, total bilirubin.

**Table 2:** Univariate and multivariate logistic regression analysis in different clinical characteristics of moderate-to-severe acute pancreatitis.

Characteristics	Comparisons	Univariate logistic		Multivariate logistic	
		OR (95% CI)	*p*-Value	OR (95% CI)	*p*-Value
LDGs	—	4.69 (2.16–10.16)	<0.001	6.36 (2.16–18.68)	0.001
Hospital day	—	1.44 (1.18–1.74)	<0.001	1.27 (0.95–1.71)	0.108
Age	—	1.00 (0.98–1.03)	0.874	0.99 (0.91–1.08)	0.856
CRP	—	1.01 (1.00–1.02)	0.009	1.02 (1.00–1.05)	0.034
NEUT	—	1.11 (1.01–1.22)	0.032	0.96 (0.74–1.23)	0.741
Gender	Male or female	0.86 (0.36–2.06)	0.728	2.05 (0.19–22.75)	0.558
Hyperlipidemia	Yes or No	1.28 (0.53–3.13)	0.586	2.36 (0.07–80.23)	0.634
Choleithiasis	Yes or No	1.48 (0.60–3.63)	0.393	1.23 (0.09–16.85)	0.878

Abbreviations: CRP, C-reactive protein; LDGs, low-density granulocytes; NEUT, neutrophil count.

**Table 3:** Predictive value of each indicators/scoring systems for acute pancreatitis to receiver operating characteristics (ROC) analysis.

Indicator	Sensitivity (%)	Specificity (%)	Cutoff	AUC (95% CI)	*p*-Value
LDGs	95.0	90.2	2.96%	0.967 (0.934–1.000)	<0.001
CRP	55.0	76.3	109.57 mg/L	0.686 (0.569–0.803)	0.005
APACHE II score	75.0	78.0	5.5	0.821 (0.728–0.914)	<0.001
BISAP score	77.5	80.5	1.5	0.845 (0.760–0.929)	<0.001

Abbreviations: APACH II score, acute physiology and chronic health evaluation; BISAP score, bedside index for severity in acute pancreatitis; CRP, C-reactive protein; LDGs, low-density granulocytes.

## Data Availability

The data that support the findings of this study are indeed available from the corresponding author upon reasonable request.

## Supporting Information

Supporting Information 1 Provides detailed scoring criteria for the APACHE II system and the BISAP score, both used to assess the severity of acute pancreatitis.

Supporting Information 2 Presents the flowchart outlining the overall study design and participant grouping.

Supporting Information 3 Includes representative flow cytometry plots displaying the proportion of peripheral blood LDGs in healthy controls (A), patients with mild acute pancreatitis (B), and patients with moderate-to-severe acute pancreatitis (C).

Supporting Information 4 Contains correlation analyses between LDGs levels and various laboratory indicators and clinical comorbidities, including WBC, glucose, calcium, NLR, APACHE II score, and BISAP score.
