# Supplementary material for: Clinical Significance of Low-Density Granulocytes in Acute Pancreatitis
Source: Mediators Inflamm. 2025 Jul 10;2025:5275081. doi: 10.1155/mi/5275081 (PMC12271696; doi:10.1155/mi/5275081)
Supplement: Supporting Information 1 — Provides detailed scoring criteria for the APACHE II system and the BISAP score, both used to assess the severity of acute pancreatitis. [file 5275081.f1.docx]

| Supplement 4.1 The APACHE II Severity of Disease Classification System | | | | | | | | | |
| --- | --- | --- | --- | --- | --- | --- | --- | --- | --- |
| Physiological Variable | +4 | +3 | +2 | +1 | 0 | 1 | 2 | 3 | 4 |
| Temperature (°C) | ≥41 | 39-40.9 | - | 38.5-38.9 | 36-38.4 | 34-35.9 | 32-33.9 | 30-31.9 | ≤29.9 |
| Mean arterial pressure (mmHg) | ≥160 | 130-159 | 110-129 | - | 70-109 | - | 50-69 | - | ≤49 |
| Heart rate (min^-1^) | ≥180 | 140-179 | 110-139 | - | 70-109 | - | 55-69 | 40-54 | ≤39 |
| Respiratory rate (min^-1^) | ≥50 | 35-49 | - | 25-34 | 12-24 | 10-11 | 6-9 | - | ≤5 |
| Oxygenation: A-aDO_2_ or PaO_2_ (mmHg) | | | | | | | | | |
| a. FiO_2_ <50% record only PaO_2_ | - | - | - | - | >70 | 61-70 | - | 55-60 | <55 |
| b. FiO_2_ ≥50% record A-aDO_2_ | ≥500 | 350-499 | 200-349 | - | <200 | - | - | - | - |
| Arterial pH | ≥7.7 | 7.6-7.69 | - | 7.5-7.59 | 7.33-7.49 | - | 7.25-7.32 | 7.15-7.24 | <7.15 |
| Serum Sodium (mmol/L) | ≥180 | 160-179 | 155-159 | 150-154 | 130-149 | - | 120-129 | 111-119 | ≤110 |
| Serum Potassium (mmol/L) | ≥7 | 6-6.9 | - | 5.5-5.9 | 3.5-5.4 | 3-3.4 | 2.5-2.9 | - | <2.5 |
| Serum Creatinine (μmol/L) | ≥305 | 172-304 | 128-171 | - | 53-127 | - | <53 | - | - |
| Hematocrit (%) | ≥60 | - | 50-59.9 | 46-49.9 | 30-45.9 | - | 20-29.9 | - | <20 |
| White blood count (×10^9^/L) | ≥40 | - | 20-39.9 | 15-19.9 | 3-14.9 | - | 1-2.9 | - | <1 |
| Glasgow Coma Scale (GCS) | Score = 15 minus actual GCS | | | | | | | | |
| Serum HCO_3_ (mmol/L) (Not preferred, use if no ABGs) | ≥52 | 41-51.9 | - | 32-40.9 | 22-31.9 | - | 18-21.9 | 15-17.9 | <15 |

A = Total acute physiology score (APS) = Sum of the 12 individual variable points. B = Age points: Assign points to age as follows: add +0 for <44, +2 for 45-54, +3 for 55-64, +5 for 65-74, and +6 for ≥75. C = Chronic health points: if the patient has a history of severe organ system insufficiency or is immune-compromised assign points as follows: a. +2 for elective postoperative patients, b. +5 for non-operative or emergency postoperative patients. APACHE II Score = A (APS) + B (Age) + C (Chronic health).

Supplement 4.2 Bedside Index for Severity in Acute Pancreatitis score (BISAP)

| one point for each criterion met |  |
| --- | --- |
| BUN＞25mg/dL (8.9mmol/L) | 1 |
| SIRS | 1 |
| Impaired mental status | 1 |
| Age＞60year | 1 |
| Pleural effusion present | 1 |

A BISAP score of ≥3 suggests progression to moderately severe acute pancreatitis (MSAP) or severe acute pancreatitis (SAP).
