# Supplementary figures and images for: Clinical Significance of Low-Density Granulocytes in Acute Pancreatitis
Source: Mediators Inflamm. 2025 Jul 10;2025:5275081. doi: 10.1155/mi/5275081 (PMC12271696; doi:10.1155/mi/5275081)

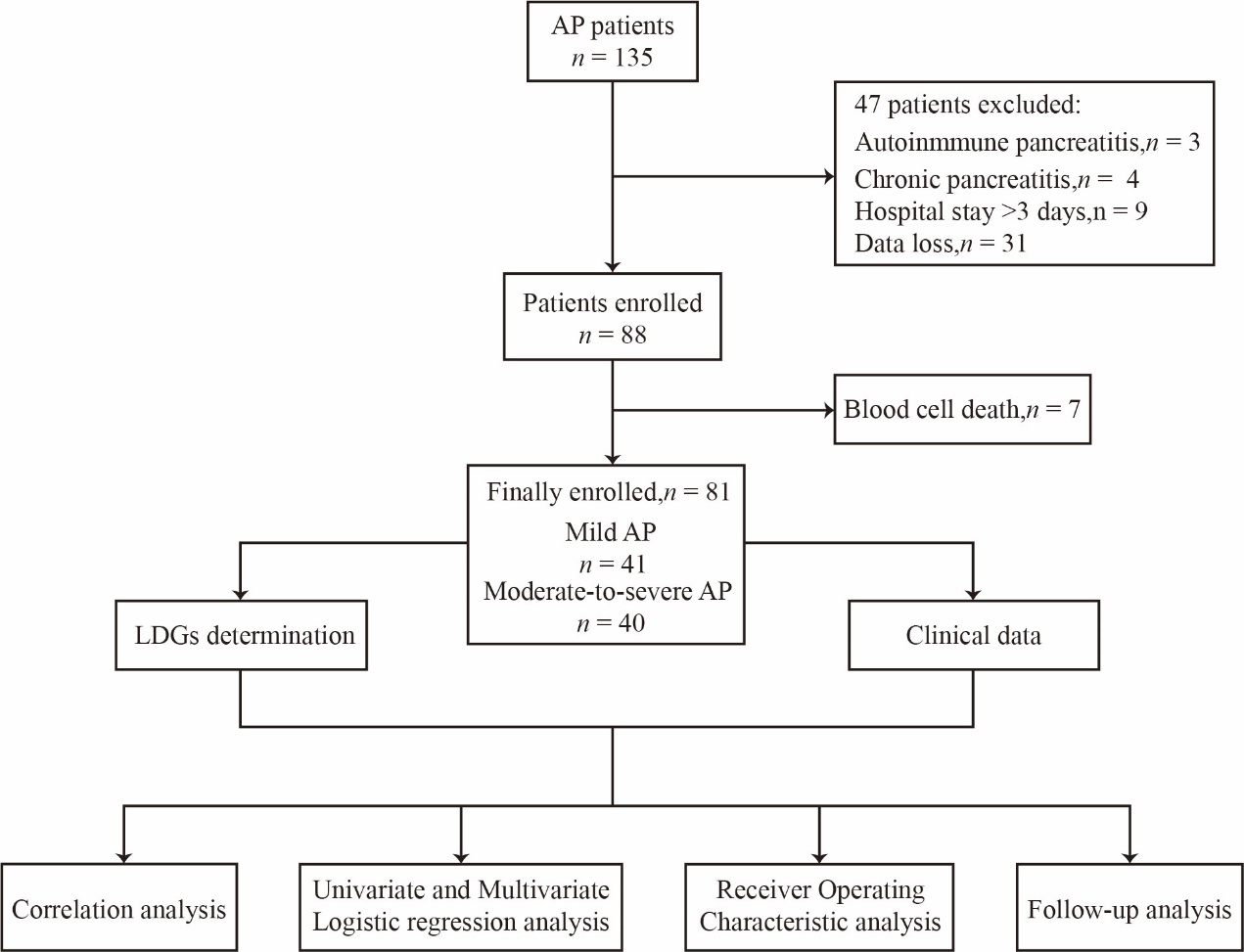


Supplement 1. Flowchart of the study

Supplement: Supporting Information 2 — Presents the flowchart outlining the overall study design and participant grouping. [file 5275081.f2.docx]
