# Supplementary material for: Clinical Significance of Low-Density Granulocytes in Acute Pancreatitis
Source: Mediators Inflamm. 2025 Jul 10;2025:5275081. doi: 10.1155/mi/5275081 (PMC12271696; doi:10.1155/mi/5275081)
Supplement: Supporting Information 3 — Includes representative flow cytometry plots displaying the proportion of peripheral blood LDGs in healthy controls (A), patients with mild acute pancreatitis (B), and patients with moderate-to-severe acute pancreatitis (C). [file 5275081.f3.docx]

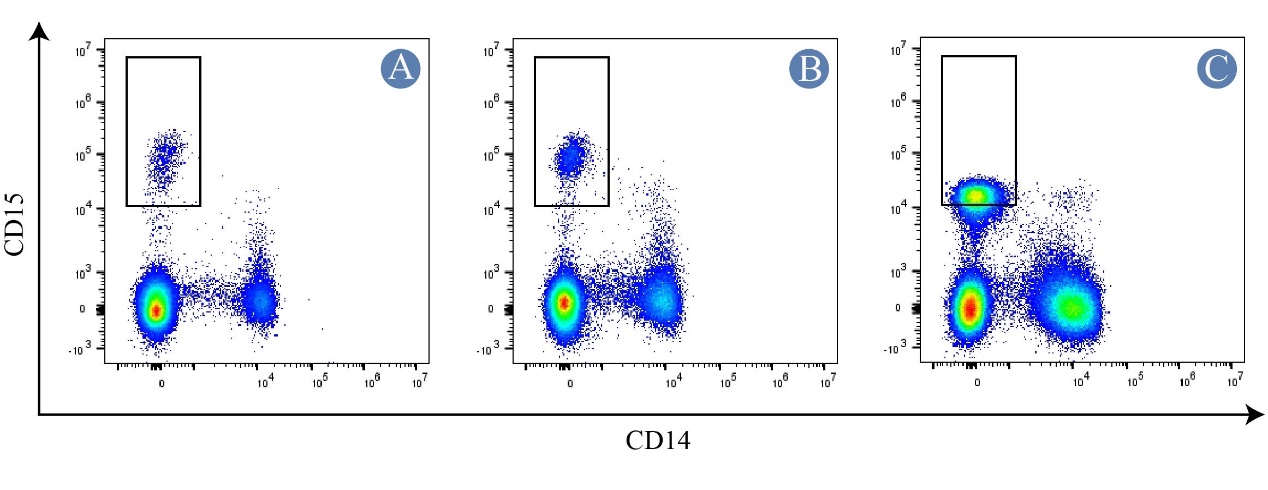


# Supplement 2(A).Displayed the proportion of peripheral blood LDGs in healthy controls (HC); Supplement 2(B).Displayed the proportion of peripheral blood LDGs in mild pancreatitis (MAP); Supplement 2(C).Displayed the proportion of peripheral blood LDGs in moderate to severe pancreatitis.
