# Supplementary material for: Clinical Significance of Low-Density Granulocytes in Acute Pancreatitis
Source: Mediators Inflamm. 2025 Jul 10;2025:5275081. doi: 10.1155/mi/5275081 (PMC12271696; doi:10.1155/mi/5275081)
Supplement: Supporting Information 4 — Contains correlation analyses between LDGs levels and various laboratory indicators and clinical comorbidities, including WBC, glucose, calcium, NLR, APACHE II score, and BISAP score. [file 5275081.f4.docx]

Supplement 3.

| Correlation analysis of laboratory indicators and comorbidities with LDGs levels | | | |
| --- | --- | --- | --- |
| characteristics | comparisons | Levels of LDGs in peripheral blood of AP patients | |
|  |  | r | *P-value* |
| WBC |  | 0.254 | 0.022 |
| CRP |  | 0.203 | 0.075 |
| PLT |  | 0.062 | 0.584 |
| ALB |  | -0.130 | 0.248 |
| TBIL |  | 0.052 | 0.644 |
| ALT |  | -0.195 | 0.081 |
| GLU |  | 0.306 | 0.005 |
| BUN |  | 0.120 | 0.288 |
| K |  | -0.147 | 0.192 |
| Ca |  | -0.338 | 0.003 |
| NLR |  | 0.366 | 0.001 |
| BMI(kg/m²) |  | 0.120 | 0.288 |
| BISAP score |  | 0.540 | ＜0.001 |
| APACHE II score |  | 0.551 | ＜0.001 |
| Hyperlipidemia | Yes vs No |  | 0.133 |
| Choleithiasis | Yes vs No |  | 0.463 |
| Alcohol | Yes vs No |  | 0.914 |
| Diabetes | Yes vs No |  | 0.333 |
| Local Complication | Yes vs No |  | 0.001 |
| ICU | Yes vs No |  | 0.095 |

WBC**,** White Blood Cell Count; CRP, C-reactive protein; PLT**,** platelet count; ALB, Albumin; TBIL, Total Bilirubin; ALT, Alanine Aminotransferase; GLU, glucose; BUN, Blood Urea Nitrogen; K, potassium; Ca, calcium; NLR**,** Neutrophil-to-Lymphocyte Ratio; BMI, Body mass index; BISAP score**,** bedside index for severity in acute pancreatitis; APACHE II score**,** Acute Physiology and Chronic Health Evaluation; ICU, Intensive Care Unit.
